# Refractometric Detection of Adulterated Milk Based on Multimode Interference Effects

**DOI:** 10.3390/foods11081075

**Published:** 2022-04-08

**Authors:** Yadira Aracely Fuentes-Rubio, Yamil Alejandro Zúñiga-Ávalos, José Rafael Guzmán-Sepúlveda, René Fernando Domínguez-Cruz

**Affiliations:** 1 Centro de Innovación Tecnológica en Eléctrica y Electrónica, Universidad Autónoma de Tamaulipas, Carr. a San Fernando Cruce con Canal Rodhe S/N. Col Arcoiris, Reynosa 88779, Tamaulipas, Mexico; yfuentes@docentes.uat.edu.mx (Y.A.F.-R.); a2173720198@alumnos.uat.edu.mx (Y.A.Z.-Á.); 2 CINVESTAV Unidad Monterrey, Vía del Conocimiento 201, Parque de Investigación e Innovación Tecnológica km 9.5 de la Autopista Nueva al Aeropuerto, Apodaca 66600, Nuevo León, Mexico; jose.guzmans@cinvestav.mx

**Keywords:** milk, adulteration, fiber optics sensor, multimode interference

## Abstract

This paper reports on the refractometric detection of water-adulterated milk using an optical fiber sensor whose principle of operation is based on multimode interference (MMI). The device is manufactured in a simple way by splicing a segment of coreless multimode fiber (NC-MMF) between two single-mode fibers (SMFs); neither functionalization nor deposition of a sensing material is required. MMI takes place in the NC-MMF and, when fed with a broadband spectrum, a transmission peak appears at the output of the MMI device due to its inherent filter-like response, whose position depends on the effective refractive index (RI) of the medium surrounding the NC-MMF. Therefore, when the sensor is immersed in different milk–water mixtures, the peak wavelength shifts according to the RI of the mixture. In this way, adulterated milk can be detected from the wavelength shift of the transmission peak. The system was tested with two commercial brands of milk, and adulterations were clearly distinguished in both cases. In the range of interest, from no dilution up to 50% dilution, the sensor exhibits a linear response with a sensitivity of −0.04251 and −0.03291 nm/%, respectively, for the two samples tested. The measurement protocol is repeatable and allows for locating the peak wavelength within <0.34 nm over several repetitions using different samples with the same concentration. A thermal sensitivity of 0.85 nm/°C was obtained, which suggests that the temperature needs to be maintained as fixed during the measurements. The approach presented can be extended to other scenarios as a quality control tool in beverages for human consumption, showing the advantages of simple construction, high sensitivity, and the potential for real-time monitoring.

## 1. Introduction

Food safety monitoring is of critical importance to human health and well-being, and lately, it has also become a major aspect of adequate nutrition strategies owing to its direct impact on public health [1,2,3]. In this area, food technologists and health scientists look for simple, rapid, and sensitive methods for assessing the status and composition of foods and beverages as well as detecting foodborne pathogens with high specificity. Common approaches involve electrochemical [4] or biochemical [5] sensors, with special focus on microbial contamination management [6]. In recent years, the trend has been toward making low-cost, easy-to-use sensing devices to perform rapid single-point measurements that can be useful for in-line assessment [7]. In this regard, photonic devices arise as a natural alternative due to its potential for non-invasive, real-time measurements [8,9,10].

Milk consumption is essential for the human diet, as it provides several nutrients, such as proteins, carbohydrates, minerals, and vitamins [11]. According to the Food and Agriculture Organization of the United Nations, both milk production and consumption have increased worldwide, mainly in developing countries [12]. As a consequence of such an increase in competition and complex supply chains, it has become common to find adulterated milk in the market [13]. The alteration of milk content can be carried out at different levels and for different purposes, for instance, adulteration of the nitrogen content, milk fat content and detergents, addition of water and other substances for dilution, and adulteration to increase the shelf life. Because of simplicity and availability, water is the most widely used adulterant [14], although it reduces its nutritional value. Additionally, if chemicals or pathogens contaminate the water, it represents a potential threat to consumers’ health. Consequently, it is a priority to have tools that allow for detection of its alteration in a simple, fast, and effective way.

Due to its relevance, several techniques have been reported besides the widespread Abbe refractometry for detecting water-adulterated milk, such as freezing point osmometry [15], digital image processing combined with chemometric tools [16], infrared spectroscopy [17,18], including its combination with chemometric approaches [19], and humidity sensors based on microwave absorption [20]. These techniques can provide valuable, detailed information; however, they do not operate in situ or in real time, and they often require complex sample preparation procedures or expensive peripherical instrumentation.

In this regard, fiber optics sensors (FOS) have received significant attention in different areas of science and technology due to their well-known advantages over conventional sensors, such as small size, fast response, immunity to electromagnetic interference, remote and on-site sensing, and its resistance to harsh environmental conditions [21]. In general, FOS have been widely exploited for measuring physical and chemical parameters in many different scenarios [22]. In the particular case of milk, for instance, FOS have been reported for the detection of formaldehyde as an adulterant [23]. The sensor was constructed by placing a water-insoluble layer of polyoxometalate salt on the optical fiber. The detection mechanism is based on spectral changes in the UV–Vis region when the coated fiber is in contact with formaldehyde.

In this paper, the refractometric detection of water-adulterated milk using a FOS is reported, in which the principle of operation is based on multimode interference (MMI) effects. The sensor’s architecture is simple and consists of a segment of a coreless multimode fiber (NC-MMF) spliced between two standard single-mode fibers (SMFs). The fabrication does not require functionalization procedures or deposition of sensing materials. In this structure, the medium surrounding the NC-MMF plays the role of its cladding, thus making the interference of multiple modes taking place in the NC-MMF to be sensitive to variations in the external refractive index (RI). In this way, changes in the RI of the aqueous medium surrounding the NC-MMF are reflected in the sensor’s spectral response such that milk adulterations can be detected and quantified with standard optical equipment. Despite several MMI-based fiber optics, refractometers have been reported in the literature; the present work entails a novel application to the food industry for the detection of milk adulterations. Moreover, to the best of our knowledge, this is the first time that MMI-based refractometry is performed on heterogeneous liquids. The proposed sensor was tested on commercial milk samples, exhibiting a linear response with respect to the adulteration level. In addition, the sensing scheme has a simple construction, and it has the potential for low-cost, real-time sensing, which can be desirable for in-line analysis.

## 2. Principle of Operation

**Figure 1:**
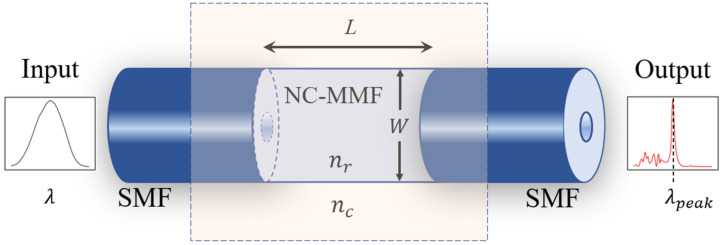
SMS structure of the proposed sensor where a section of coreless multimode fiber (NC-MMF) is spliced between two standard single-mode fibers (SMFs). The input and output spectra depict the characteristic spectral filtering of an MMI device resulting from the interference of multiple modes taking place in the NC-MMF, where constructive interference is achieved only for some wavelengths. For a fixed length of NC-MMF, variations in the location of the peak wavelength can be associated directly with changes in the RI of the medium surrounding the NC-MMF (see text for details).

The structure of the proposed sensor, shown in Figure 1, is a particular case of the more general singlemode–multimode–singlemode (SMS) architecture, which consists of a segment of a multimode fiber (MMF) spliced between two single-mode fibers (SMFs) [24,25,26]. In the present case, the fiber used is a special type of MMF that is coreless (NC-MMF), such that the medium around the NC-MMF plays the role of its cladding. This type of arrangement provides a large sensitivity to the fiber surroundings in a straightforward manner while keeping the sensor’s architecture and a simple fabrication [27].

In general, when light propagates from the SMF to the MMF, several optical modes are excited and interfere with one another as they propagate along the MMF, giving rise to a characteristic interference pattern for each wavelength [26]. As a result, the input field can be replicated (self-imaging) periodically at certain propagation distances. When another SMF is placed at the output of a segment of MMF of fixed length and a broad spectrum is fed into the MMI device, only a specific wavelength satisfies a constructive condition at the output [26].

As a result of this filter-like response, the peak wavelength, λ*_peak_*, that replicates the *p*-th image of the input field in an MMI device of length *L* is [28,29]:

(1) $$\lambda_{peak}=p\frac{n_{eff}W_{eff}^{2}}{L}$$

where *p* = 1,2,3,… is the order of input field self-image; $n_{eff}$ and $W_{eff}$ are the effective refractive index (RI) and effective optical diameter of the MMF, respectively. $W_{eff\;}$ accounts for the (polarization-dependent) penetration depth into the cladding beyond the geometrical diameter, W, associated with the Goos-Hanchen shift at the boundary between the core and the cladding.

Strictly, both $n_{eff}$ and $W_{eff}$ are mode dependent. However, the MMI theory allows for simplifications based on the characteristics of the lower-order modes that propagate in the MMF [28,29,30]. For instance, $n_{eff}$ and $W_{eff}$ are typically associated with the fundamental mode and the so-called beating length, from where Equation (1) is derived, which is calculated based on the two lowest-order modes [28,29,30]. Taking these simplifications and based on the cylindrical geometry of the MMF, $W_{eff}$ can be calculated by averaging the penetration of the fundamental mode for the two orthogonal polarizations [28,29]:

(2) Weff=W+12λ0πnr2−nc2−12ncnr2+1

where W is the geometrical diameter of the MMF; $\lambda_{0}$ is the free-space wavelength; $n_{r}$ and $n_{c}$ is the RI of the core and the cladding of the MMF, respectively. Note that, strictly, $n_{r}$, $n_{c}$, and $W_{eff}$ are implicit functions of $\lambda_{0}$. Similarly, $n_{eff}$ can be estimated from the propagation constant of the fundamental mode knowing that, in general, the propagation constant of the *m*-th mode can be estimated as [28,29,30]:

(3) $$\beta_{m}\approx k_{0}\left[n_{r}-\frac{n_{r}}{2}{\left(\frac{\left(m+1\right)\pi }{k_{0}n_{r}W_{eff}}\right)}^{2}\right]$$

where the term in the square brackets can be regarded as the effective RI of the *m*-th mode, i.e., $\beta_{m}=k_{0}n_{eff,m}$. For the fundamental mode m=0.

To illustrate the capability of the proposed structure to detect milk adulterations, we simulated the MMI device shown in Figure 1 for operation under self-imaging conditions, at p=4, and for the case when the RI of the medium surrounding the NC-MMF, $n_{c}$, is variable. The diameter of the NC-MMF is W=125 μm, its length is L=58.23 mm, and its RI, $n_{r}$, was taken as that of fused silica. At room temperature and at a free-space wavelength of $\lambda_{0}=1560\;\mathrm{nm}$, $n_{r}=1.444024$ [31,32]. For the calculations, $n_{c}$ varied in the range from 1.00 to 1.35. These limits represent the baseline condition when the NC-MMF is surrounded by air and the upper limiting case when the NC-MMF is immersed in milk. Based on a literature review summarized in Appendix A, an RI value of 1.35 is reasonable for samples of commercial milk.

**Figure 2:**
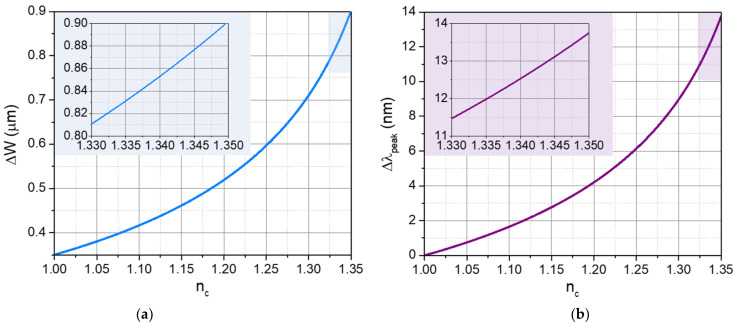
(**a**) Evanescent penetration beyond the geometrical diameter of the NC-MMF (second term in Equation (2), labeled as ΔW) as a function of $n_{c}$. (**b**) Wavelength shift with respect to a baseline condition where the NC-MMF is surrounded by air as a function of $n_{c}$. The insets show in more detail the range of interest for water–milk mixtures (see text for details).

Figure 2a shows the evanescent penetration beyond the geometrical diameter of the NC-MMF, the second term in Equation (2), which we have labeled as ΔW, as a function of $n_{c}$. This evanescent interaction is the main underlying origin of the sensing capabilities. The inset shows in more detail the shaded region from $n_{c}=1.33$ to $n_{c}=1.35,$ whose limits correspond approximately to the NC-MMF being surrounded by water and milk, respectively.

For many practical situations, e.g., high RI contrast between the core and the cladding or MMFs with a diameter much larger than the wavelength, ΔW represents a correction smaller than one wavelength, as it is also our case (see Figure 2a). Moreover, we noticed that $n_{eff}$ (not plotted) changes only in the sixth decimal digit in the entire range of $n_{c}$. Therefore, it is common to find in the literature that the approximations $n_{eff}\approx n_{r}$ and $W_{eff}\approx W$ hold as a good estimate [26].

Figure 2b shows the corresponding wavelength shift, using Equations (1)–(3), with respect to a baseline condition where $n_{c}=1.00$, i.e., when the NC-MMF is surrounded by air. The inset shows the range from $n_{c}=1.33$ to $n_{c}=1.35$ in more detail. From these results, the MMI peak wavelength spans around 2 nm for water–milk systems (inset of Figure 2b), which is resolvable with commercial-grade optical spectrum analyzers, whose typical resolution is <0.1 nm. In this simple way, the spectral shift of the SMS device’s response can be used for the refractometric detection of water-diluted milk.

## 3. Materials and Methods

### 3.1. Sample Preparation

Binary water–milk mixtures were prepared using deionized water (^®^Sigma Aldrich, San Louis, MO, USA, purity of 99%) and commercial-grade homogenized milk (purity information not available). The mixtures were prepared using two Mexican commercial brands of evaporated milk, labeled as A and B, in the range from 10 to 50% *v*/*v*, with increments of 10%. The dilution was made by volume fraction (% *v*/*v*) [33].

### 3.2. Sensor Fabrication and Engineering Considerations

The MMI sensor was fabricated using SMF-28 fiber (^®^Thorlabs, Newton, NJ, USA), which has a cladding and core diameter of 125 and 8 µm, respectively, and NC-MMF (FG125LA, ^®^Thorlabs, Newton, NJ, USA) with RI of $n_{r}=1.4445$ and a diameter of W=125 μm. Commercial FC/PC connectors were spliced at the ends of the SMS structure. The fibers were spliced using a commercial-grade arc fusion splicer (Fujikura, San Jose, CA, USA, model FSM-60S). The sensor’s fabrication requires neither functionalizing nor depositing of a sensing material on the surface of the NC-MMF. The NC-MMF was used as purchased; only the polymer coating was removed to expose the silica glass rod.

As expressed in Equation (1), the peak wavelength of the spectral response of an MMI device depends on several parameters (p, $n_{eff}$, $W_{eff}$, and L). Therefore, $\lambda_{peak}$ does not have an absolute value that is characteristic of a particular substance, but it can be adjusted to fall within the spectral window of interest depending upon the necessities of the sensing application. All parameters need to be considered to engineer the MMI device. The engineering procedure is as follows. First, the spectrum of the broadband light source needs to be considered. In the present case, a superluminescent diode was used whose emission spectrum moves from 1440 to 1680 nm. These light sources typically exhibit a Gaussian-like emission spectrum with their maximum intensity at the middle of the spectrum, 1560 nm in the present case. One typically operates around the wavelength of maximum intensity owing to better light budget. Next, it was determined that the best operation mode was at the fourth self-image (p=4) due to its associated narrower spectral response, which makes it easier to follow small spectral shifts [26]. The NC-MMF available in our lab has a diameter of W=125 μm, and it consists of a solid silica glass rod without any doping material ($n_{r}=1.4445$); these parameters roughly determine the value of $W_{eff}$ and $n_{eff}$, respectively. Then, the range of RI of the cladding, $n_{c}$, needs to be accounted for to better approximate the value of $W_{eff}$ and estimate the corresponding range for the spectral shift, $\lambda_{peak}$. In the present case, the extremes of the range for $n_{c}$ correspond to the RI of water and undiluted milk, whose RI was taken from the exhaustive literature review reported in Appendix A. With all these design restrictions, the free parameter to adjust the spectral window of operation is the length of the MMI device, L. Thus, by carefully cleaving the NC-MMF at the right length, the MMI device can be designed to operate within the spectral window of interest, typically around the maximum intensity of the broadband light source. Under these conditions, according to Equation (1), a length of L=57.8 mm gives $\lambda_{peak}\approx 1560\;\mathrm{nm}$ for the baseline condition where the MMI device is in the air.

### 3.3. Experimental Set-Up

**Figure 3:**
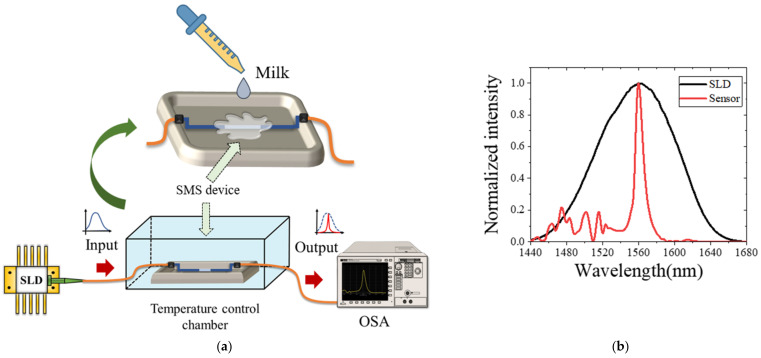
(**a**) Experimental set-up for water-adulterated milk detection. (**b**) The spectrum of the SLD light source and the SMS sensor.

The measurements were performed using the experimental set-up shown in Figure 3a. The MMI sensor was fixed in a container where the sample was deposited. The input end of the sensor was connected to a broadband light source (compact superluminescent diode, SLD1550S-A1, bandwidth 1440–1680 nm, ^®^Thorlabs) operated with a temperature controller (CLD1015, ^®^Thorlabs). The output end of the sensor was connected to an optical spectrum analyzer (OSA, Anritsu MS9740A) to acquire the transmitted signal. The inset of Figure 3a shows the NC-MMF surrounded by the sample under test. A temperature control chamber was used to prevent thermal fluctuations in the fiber device. All measurements were carried out at 25 °C. In Figure 3b, we show the normalized spectrum at the output of the MMI sensor when the NC-MFF is in the air. For reference, we also plot the input spectrum to illustrate the sensor’s filter-like spectral response.

## 4. Results and Discussion

With the MMI sensor fixed in the container, the measurements were carried out by immersing it in each milk–water mixture such that the NC-MMF is completely surrounded by the sample under test. For each sample, the output spectrum was recorded using the OSA. After each measurement, the sensor was cleaned using deionized water and was air-dried to leave it at the same initial conditions for the next sample. A similar process was followed for both milk brands.

**Figure 4:**
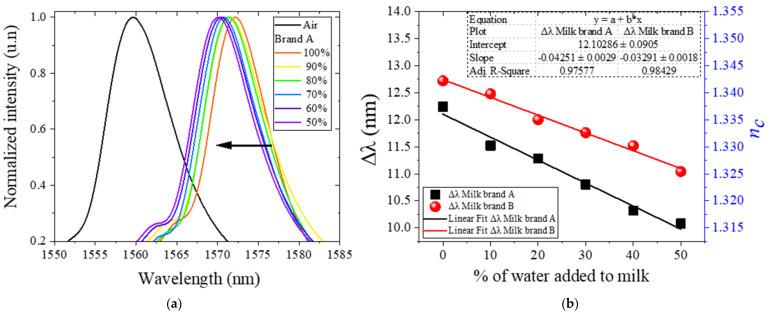
(**a**) Spectral response of the MMI sensor for water-adulterated blends for brand A. For reference, the spectral response at the baseline condition (when the sensor is in the air) is also plotted. (**b**) The spectral shift of the peak wavelength, with respect to the baseline condition, as a function of the amount of water added to the sample, for the two brands tested.

As mentioned in Section 2, variations in the RI of the medium surrounding the NC-MMF change the interference conditions and result in a spectral shift. In Figure 4a, we show the spectral response when the MMI sensor is immersed in the samples set for brand A. As expected from the simulations (Figure 2b), the spectral response associated with water–milk mixtures is shifted around 10–13 nm with respect to the baseline condition when the sensor is in the air.

Moreover, if in Figure 4a, one takes the spectrum of the undiluted sample as reference, the spectra for the diluted samples suffer a negative spectral shift that is proportional to the dilution level. This characteristic behavior is due to the fact that water has a lower RI than milk; thus, the effective RI of the water–milk mixture decreases with an increasing amount of water present in the dilution.

In Figure 4b, we summarize the spectral shift of the peak wavelength, $\Delta \lambda_{peak}$, with respect to the baseline condition when the sensor is in the air, as a function of the amount of water added to the sample, for the two brands tested. As expected from the simulations (Figure 2b) and the observations in Figure 4a, the spectrum shifts linearly with respect to the undiluted sample according to the dilution level. By fitting the experimental data to a linear function, the sensitivity obtained is −0.04251 and −0.03291 nm/% for brand A and B, respectively, as indicated in Figure 4b.

Moreover, based on the maximum instrument resolution of the OSA used in our experiments (0.03 nm), the sensitivities obtained indicate that adulterations of the sample as small as −0.705% and −0.911% could be detected for brand A and B, respectively. In other words, variations in the dilution level in the order of 1% can be monitored. This suggests that our approach could be suitable for in-line quality control of milk production.

The results shown in Figure 4b reveal further details. First, inter-brand variations can be clearly detected (even for undiluted samples), which indicates the sensor’s sensitivity to different product formulations. This sensitivity to different formulations is further verified by noting that the dilution of the two brands results in a linear spectral shift, but the slopes are different. This could be attributed to the distinct preparation process of milk and could be indicative of the mixing efficiency with water. Disclosing such a level of detail requires more delicate experiments where the formulation can be changed controllably. Nevertheless, the important conclusion for the purposes of this work is that the MMI sensor can clearly detect intra-brand variations, that is, alterations of a milk sample with respect to its undiluted state.

Moreover, the effective RI of the water–milk mixture can be retrieved from the spectral shift, as indicated by the secondary axis in Figure 4b. In a simple way, the value of $n_{c}$ can be found from the simulations shown in Figure 2b by simply taking the value of $n_{c}$ that produces the measured spectral shift. This simple approach is consistent because the spectral shift both in the simulations and the experiments is measured with respect to the same baseline where the NC-MMF is surrounded by air. Even if the simulations presented here were not available, Equations (1)–(3) can be used to retrieve the value of $n_{c}$ corresponding to a certain spectral shift. From this analysis, the RI of the water–milk mixtures is in the range from 1.3147 (50%) to 1.3374 (0%) for brand A, and from 1.3256 (50%) to 1.3417 (0%) for brand B, respectively, which agrees well with the values reported in the literature (see Appendix A).

**Figure 5:**
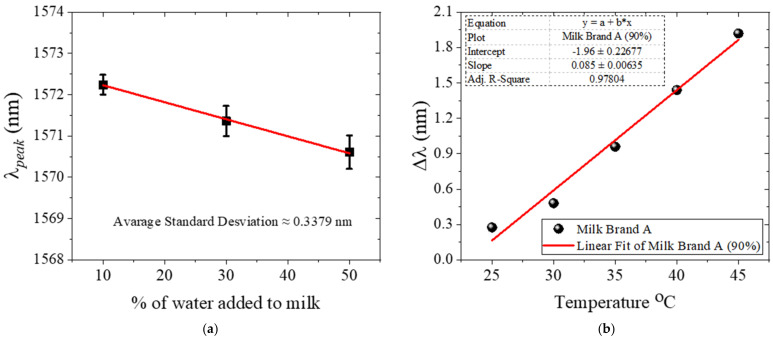
(**a**) Wavelength peaks measured for three representative water–milk mixtures (10/90, 30/70 and 50/50, respectively). Each wavelength reading was performed three times to verify the repeatability of the experiment. (**b**). Thermal effect measured at a concentration of 90% milk of brand A. Measurements were performed in the temperature range from 25 to 45 °C in increments of 5 °C.

We also studied the errors and repeatability of the measurements, for which we selected representative samples with milk brand A in concentrations of 90%, 70%, and 50%. For each of the samples selected, the wavelength peaks were measured and repeated three times following the same process described above, including washing, cleaning, and drying of the sensor. These wavelength peaks are plotted in Figure 5a. The average standard deviation of all these repetitions is approximately 0.3379 nm.

Thermal effects were also explored. For this, measurements were performed at different temperatures using the sample with 90% milk concentration of brand A. After covering the MMI sensor with the sample, the container was placed on a hot plate (^®^IKA, Wilmington, NC, USA, Model HS7), and the temperature was increased from 25 to 45 °C in increments of 5 °C. The whole arrangement was kept inside the temperature chamber. At each temperature, the sample was stabilized for 20 min before recording the spectrum. The results are summarized in Figure 5b. A thermal sensitivity of 0.85 nm/°C was obtained, which indicates that the temperature needs to be kept constant during the measurements to avoid masking the small variations of the RI. In this regard, simple strategies exist to athermalize MMI devices, such as partially covering the NC-MMF with a polymer coating in which the thermo-optic coefficient compensates for the thermal effects of silica [34,35,36]; nevertheless, in this case, the large thermal sensitivity originates from the sample itself, most likely due to the milk’s fat content, which makes it imperative to reduce temperature fluctuations during the measurements. Finally, in the present, the humidity in the chamber was not controlled because (i) the volume of the sample is large (1 mL), (ii) the measurement time per sample is short (less than 10 min), and (iii) the measurement is based on the evanescent interaction between the light and the surrounding liquid, which takes place practically on the surface of the NC-MMF. In these conditions, evaporation or wetting effects are negligible. However, in other conditions, e.g., small sample volumes, humidity is required to be monitored and controlled during the measurements.

## 5. Conclusions

In summary, in this paper, a practical approach was presented for detecting water-based adulterations of milk. For this, an MMI fiber optics sensor was used with a simple architecture consisting of a section of coreless MMF spliced between two standard SMFs. The sensing scheme was tested on two commercial milk brands, which were controllably diluted with water. Sensitivity in the order of –(0.033–0.042) nm/% was obtained for the brands tested, which, in turn, indicates that small dilutions in the order of 1% can be detected with the OSA used. Moreover, even for undiluted samples, the sensor can clearly discriminate different brands, indicating the sensitivity to inter-brand variations in the formulation of milk. The results also reveal a large thermal sensitivity, most likely due to the fat content in milk, which could affect the sensor’s capability to detect small RI variations if the temperature is not kept constant during the measurements.

Overall, the results presented indicate that the MMI-based sensing approach constitutes a suitable alternative both for the detection of milk adulterations and for the in-line quality control assessment in milk production factories. The sensor has a simple architecture, and it can be fabricated easily with standard equipment, i.e., commercial arc fusion splicer. Moreover, neither functionalization nor deposition of sensing materials is required. The sensor’s fabrication has the potential to be low cost because it is based on commercial-grade conventional fibers. Finally, despite that the inherent sensitivity is high enough, using some coatings, e.g., high-RI films or plasmonic layers, could help to increase the sensitivity even further, if necessary.

## Data Availability

Not applicable.

## Appendix A

**Reference**	**Year**	**RI**	**Notes**
[37]	1947	1.3449–1.4480 1.3461–1.3500	More than 200 samples of cow and buffalo raw milk were tested over a period of 8 months, with an Abbe refractometer, at 40 °C. The RI limits reported in this table are for cow milk (mode is 1.3463) and buffalo milk (mode is 1.3480), respectively.
[38]	1999	$n_{eff}=1.3326+1.91803\times 10^{-4}C$	The RI of milk diluted with distilled water was measured with both a reflectometer and an Abbe refractometer. It is not specified if milk is commercial or raw. The expression in this table was estimated from fitting the RI reported as a function of milk concentration to a straight line (C is in %, it takes values from 0 to 100; R2 > 0.99).
[39]	2001		The RI of commercial milk samples was measured with a reflectometer. The samples consisted of untreated milk with fat volume concentrations of 0.004%, 1.53%, and 3.55%. Three different methods were used: A: reflectometry; B: reflectometry at a critical angle; C: surface plasmon resonance. For each method, the RI values are given for the different fat concentrations.
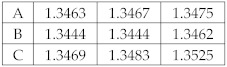
[40]	2010		The RI and attenuation coefficient were measured simultaneously from reflectance measurements. The samples consisted of commercial milk (with fat volume concentrations ≤3.3%), and milk–cream mixtures, without any dilutions of these samples. In this table, only the real part of the complex RI is reported; the first column corresponds to measurements performed with a commercial Abbe refractometer.
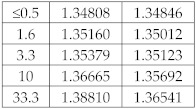
[41]	2015	1.3440–1.3485	RI range given for cow milk at 20 °C and 589 nm, measured with an Abbe refractometer. It is not specified if the milk is commercial or raw. It is also reported that the RI of milk fat is usually in the range of 1.4537 to 1.4552 at 40 °C.
[42]	2019	1.3425–1.3570	Commercial milk was used (toned, standardized, and full cream with 3%, 4.5% and 6% of fat, respectively) as well as fresh unpasteurized cow and buffalo milk. All experiments were carried out at room temperature using a commercial refractometer. The RI range reported is from 50% dilution with DI water to undiluted milk; it is not specified on which samples the measurements were performed.
[43]	2020	1.3445–1.3487	The RI of 240 raw milk samples was measured throughout one year (average value of 1.3461). The average RI is also given per season: spring (1.3465), summer (1.3455), autumn (1.3458), and winter (1.3468). It is not stated how the RI measurements were performed.
